# The global cancer genomics consortium's symposium: new era of molecular medicine and epigenetic cancer medicine - cross section of genomics and epigenetics

**Published:** 2015-01

**Authors:** Masakazu Toi, M. Radhakrishna Pillai, Sudeep Gupta, Rajendra Badwe, Maria Carmo-Fonseca, Luis Costa, Louis WC Chow, Stefan Knapp, Rakesh Kumar

**Affiliations:** ^1^ Kyoto University Graduate School of Medicine, Kyoto, Japan; ^2^ Organisation for Oncology and Translational Research, Kyoto, Japan; ^3^ Rajiv Gandhi Center for Biotechnology, Thiruvananthapuram, India; ^4^ Tata Memorial Centre, Advanced Centre for Treatment, Research and Education in Cancer, Mumbai, India; ^5^ Institute of Molecular Medicine, Lisbon, Portugal; ^6^ Hospital de Santa Maria – CHLN, Lisbon, Portugal; ^7^ Structural Genomic Consortium, University of Oxford, Oxford, UK; ^8^ Department of Biochemistry and Molecular Medicine, School of Medicine and Health Sciences, George Washington University, Washington, DC, USA

**Keywords:** Transcriptome, Epigenetics, Cancer Stem Cells, Tumor Heterogeneity, Isogenic Clones, Tumor Biology

## Abstract

The Global Cancer Genomics Consortium (GCGC) colleagues continue to function together as an interactive multidisciplinary team of cancer biologists and oncologists with interests in genomics and building a bidirectional bridge between cancer clinics and laboratories while taking advantage of shared resources among its member scientists. The GCGC includes member scientists from six institutions in Lisbon, United Kingdom, Japan, India and United States, and was formed in December 2010 for a period of five years. Driven by valuable lessons learned from the previous symposiums, the fourth GCGC Symposium focused on a cross section of genomic and epigenetic cancer medicine and it's for this reason we chose the conference theme - New Era of Molecular Medicine and Epigenetic Cancer Medicine: Cross Section of Genomics and Epigenetics. This year's symposium was co-organized by the Organization for Oncology and Translational Research (OOTR) at the Shiran Hall, Kyoto University, Kyoto, Japan, from November 14 and 15, 2014. The symposium attracted around 80 participants from 14 countries, and counted with 23 invited platform speakers. Scientific sessions included eight platform sessions and one poster session, and three plenary lectures. The symposium focused on cancer stem cells and self-renewal, cancer transcriptome, tumor heterogeneity, tumor biology, breast cancer genomics, targeted therapeutics and personalized medicine. The issues of cancer stem cells and tumor heterogeneity were echoed in most of the scientific presentations. The meeting concluded with an oral presentation by the best poster awardee and closing remarks by meeting co-chairs.

## INTRODUCTION

The Global Cancer Genomics Consortium (GCGC) colleagues continue to function together as an interactive multidisciplinary team of cancer biologists and oncologists with interests in cancer genomics and building a bidirectional bridge between cancer clinics and laboratories while taking advantage of shared resources among its member scientists. The GCGC includes member scientists from six institutions in Lisbon, United Kingdom, Japan, India and United States, and was formed in December 2010 for a period of five years. Since the last successful Symposium in Lisbon, Portugal in 2013, the GCGC colleagues continue to function together as an interactive multidisciplinary team of cancer biologists and oncologists with interests in genomics and building a bidirectional bridge between cancer clinics and laboratories while taking an advantage of shared resources among its founding members. This collaborative network continues to enable cancer students and scholars in academics and research and helped to nurture a cross-disciplinary collaborative approach among consortium members and meeting participants over the years.

Motivated by valuable lessons learned from the previous symposiums, the fourth GCGC Symposium focused on a cross section of genomic and epigenetic cancer medicine. And it's for this reason we chose the conference theme - New Era of Molecular Medicine and Epigenetic Cancer Medicine: Cross Section of Genomics and Epigenetics. This year's symposium was co-organized by the Organization for Oncology and Translational Research (OOTR) at the Shiran Hall, Kyoto University, Kyoto, Japan, from November 14 and 15, 2014. The symposium attracted around 80 participants from 14 countries, and counted with 23 invited platform speakers and a rich poster session.

Similar to previous years, the mission of the symposium was to provide practical knowledge on structural and cancer genomics approaches, to meet and share experiences of the like-minded cancer experts and leaders, to provide an exclusive platform for focused cancer genomics endeavors, and to evaluate the latest research developments in cancer genomics and epigenetics.

### Focus on the Molecular Medicine and Epigenetic Cancer Medicine

The fourth GCGC meeting was at the interface of molecular cancer medicine, epigenetics and tumor biology with a particular focus on biologic questions within recognizable clinical framework with a forward looking approach. The event was opened by Dr. Masakazu Toi with a brief history of GCGC. Dr. Toi introduced the chief guest Prof. Nagahiro Minato, Executive Vice-President for Research, Planning, and Hospital Administration at the Kyoto University. Prof. Minato inaugurated the meeting by highlighting the contribution of genome research in cancer biology and treatment and the role played by the Kyoto University in the previous consortium meetings. He stressed the need to developing efficient collaborative programs to understand the molecular and genomic details of human cancer and to develop targeted cancer therapeutic. Scientific sessions included eight platform sessions and one poster session, and included three plenary lectures. The symposium focused on the following scientific themes: 1) cancer stem cells and self-renewal; 2) cancer transcriptome; 3) tumor heterogeneity; 4) tumor biology; 5) breast cancer genomics; 6) targeted therapeutics; and 7) personalized medicine. The issues of cancer stem cells and tumor heterogeneity are echoed in most of the scientific presentations. The meeting concluded with an oral presentation by the best poster awardee and closing remarks by meeting co-chairs - Dr. Masakazu Toi and Dr. Rakesh Kumar.

### Cancer Stem Cells and Self-renewal

The theme was introduced by the opening conference lecture by Dr. Akihiko Yokoyama from the Laboratory of Malignancy Control Research, Kyoto University School of Medicine. Dr. Yokoyama presented new molecular insights by which Mixed-lineage leukemia fusion (MLL) oncogenes aberrantly regulate the expression of hematopoietic stem genes, leading to leukemia [1, 2]. Dr. Yokoyama provided experimental evidence to suggest that MLL fusion proteins recognize its target genes through specific bindings of the PWWP domain of LEDGF and the CXXC domain of MLL that specifically bind to H3K36me2/3 and non-methylated CpGs, respectively. Because H3K36me2/3 marks are generally linked to gene activation, this suggested that previously transcribed genes with non-methylated CpGs in its promoters are likely to be stimulated by MLL fusion proteins. Dr. Yokoyama suggested that in general, MLL fusion oncogenes function as a facilitator of self-renewal simply by continuously activating previously-active hematopoietic stem cell program genes to transform hematopoietic progenitors into cancer cells.

Dr. Hiroshi Seno from the Department of Gastroenterology and Hepatology, Kyoto University Graduate School of Medicine highlighted the role of tumor stem cells in digestive tumors and presented data outlining the benefits of using lineage tracing approach in murine models in identifying tumor stem cells (TSCs) from resembling normal stem cells (NSCs) in the background of self-renewal and continuous supply of progeny tumor cells. Specifically, he utilized Dclk1creERT2/+ knock-in mice to understand the role of doublecortin-like kinase 1 (Dclk1) in the intestinal tumors. Dr. Seno's group found that Dclk1 does not mark intestinal NSCs and that targeting Dclk1-marked TSCs marked could be used an antitumor therapy without any significant damages to the normal intestines in murine models. Dr. Seno suggested that Dclk1 might be a unique marker to discriminate tumor stem cells from normal stem cells in the mouse intestine.

Dr. Nobu Oshima from the Department of Reprogramming Science, Center for iPS Cell Research and Application at Kyoto University shared exciting data showing the ability of OCT3/4, SOX2 and KLF4 to induce cancer stem cell with self-renewing characteristics in colon cancer cells. He also presented a new methodology to collect the induced CSCs based on the dye-efflux activity enhancement. Dr. Oshima judged the functionality of induced CSC by its ability to differentiate in vivo and form tumors which remarkably mimicked actual human colon cancer tissues in terms of their pathological findings - intratumor heterogeneity and glandular-like structure. Dr. Oshima hopes that these methods will contribute towards the development of better therapeutic approaches targeting CSCs.

Dr. Yoshiteru Murofushi from the Laboratory of Malignancy Control Research, Medical Innovation Center at Kyoto University Graduate School of Medicine, presented a new disease model of pheochromocytoma using VHL patient-derived iPS cells. Von Hippel-Lindau (VHL) disease is an autosomal dominant inherited, family tumor syndrome caused by a germ line mutation in the VHL tumor suppressor gene. The major problem of research for VHL disease is the lack of disease model that successfully recapitulate human disease phenotypes in vitro and in vivo. To establish novel experimental model, Dr. Murofushi's laboratory has used VHL patient-derived human iPS cells (VHL iPS) to differentiate in culture. Although the remaining wild type VHL should be disrupted to recapitulate the development of pheochromocytomas, VHL iPS cells might be useful for establishing new disease model of VHL disease, especially for pheochromocytomas.

Dr. Takayuki Kawai from the Division of Hepatobiliary, Pancreas and Transplant Surgery, Kyoto University Hospital presented data showing that Keratin 19-positive cells possess cancer stem cell properties in human hepatocellular carcinoma. Dr. Kawai's work demonstrated that FACS-isolated single K19+ cells are able to self-renew, proliferate, exhibit resistance to 5-fluorouracil, express EMT related genes and form tumors in xenograft models and many of these activities are blocked by transforming growth factor beta receptor 1 inhibitor. Interestingly, K19+ HCC patients exhibit larger tumors and shorter recurrence-free survival.

### Cancer Transcriptome

Dr. Amit Dutt from The Advanced Centre for Treatment, Research and Education in Cancer (ACTEC) shared emerging data about the status of Notch pathway in an Indian cohort of tongue squamous cell carcinoma, a sub type of head and neck cancer. The group performed DNA copy number and gene expression profiling of NOTCH signaling pathway genes comprising of ligands, receptors and target genes using quantitative real-time PCR followed by immuno-histochemical analysis of activated NOTCH. In addition to the tumor suppressor function of the Notch pathway, the data presented underlined high copy number and overexpression of NOTCH signaling pathway components, consistent with an independent TSCC cohort from the TCGA study. Dr. Dutt presented data examining the influence of NOTCH1 knockdown on the treatment of HNSCC cells with gamma secretase inhibitor XXI to achieve an effective inhibition of the growth, survival and migration of cells derived from patients of Indian origin.

Dr. Wonshik Han from the Seoul National University College of Medicine Korea used the targeted kinome sequencing on NGS platform to generate a comprehensive profiling of the most frequently altered genes and mutations in triple-negative breast cancer tissues with matched normal tissues or peripheral blood samples.

Dr. Nuno Barbosa-Morais from the Institute of Molecular Medicine highlighted the importance of alternative splicing (AS) in cancer and the opportunity provided by next-generation sequencing to profile AS in tumor samples, having shared preliminary results of analyses on RNA-Seq data for clear cell renal cell carcinomas and matched normal samples from The Cancer Genome Atlas project. It was observed that AS patterns primarily separate normal from tumor samples. Some of the measured normal/tumor isoform “switches” are markers of epithelial-mesenchymal transition (EMT). Survival analysis revealed several AS events, some reassuringly known to be associated with cancer progression, as candidate markers for prognosis, both in normal and tumor samples. Gene set enrichment analyses revealed differential expression of genes associated with oncogenic processes, namely EMT, between survival strata defined based on candidate AS events. Dr. Barbosa-Morais presentations illustrate the potential of AS signatures derived from tumor transcriptome in providing etiological leads for cancer progression and as a clinical tool.

Dr. Reshmi G from the Rajiv Gandhi Centre for Biotechnology, India, presented the utility of computational modeling approach to understand the impact of viral oncoproteins upon microRNA clusters in cancer. The goal of these integrated in-silico genomic studies to identify microRNA and transcription factor-mediated regulatory networks as early detection or prognostic markers in HPV induced cervical cancer. The group systematically explored feed-forward loops consisting of miRNAs and transcription factors and applied additive and weighted modelling to combine possible transcription factor-miRNA target predictions to identify the regulation change at a specific gene set, pathway, or interacting network. The approach successfully identified novel, candidate miRNA-TF networks, for further experimental design.

Dr. Raja Mazumdar from the George Washington University presented exciting way to conduct pan-cancer analyses because cancer mutation and expression data is distributed in multiple databases. We have integrated cancer related mutation and expression data from multiple databases and publications to create two comprehensive mutation and expression databases called BioMuta and BioExpress. The group then used these databases to identify driver mutations present in multiple cancers and identified 62 mutations in 13 genes affecting functional sites such as DNA and ATP binding and also various post-translational modifications (PTM) sites. Key driver mutations identified includes p53 acetylation and zinc binding sites, CTNNB1 phosphorylation sites and SF3B1 ubiquitylation site. Initial expression data analysis show that genes which are highly mutated in cancer usually do not show differential expression in tumors compared to adjacent normal tissue. It is hoped that the approach undertaken by Dr. Muzumdar's lab will facilitate identification of priority diagnostic and therapeutic targets.

### Tumor Heterogeneity

Dr. Seishi Ogawa, Professor of Pathology and Tumor Biology at the Graduate School of Medicine Kyoto University presented a plenary lecture on mutational landscape and clonal architecture in low-grade gliomas. Dr. Ogawa shared his experience with the pathogenesis of lower grade glioma which if not treated early on leads to devastating high grade gliomas. Despite recent findings of frequent mutations in *IDH1* and other genes, our knowledge about the pathogenesis of lower grade is still incomplete. By combining high-throughput sequencing data of 760 cases from two large cohorts with extensive validation sequencing, the team delineated the entire picture of genetic alterations and affected pathways in Lower grade gliomas with sensitive detection of novel drivers. Lower grade gliomas are classified into three distinct subtypes characterized by discrete sets of mutations and distinct clinical behaviors. Mutations showed significant positive/negative correlations and chronological hierarchy as inferred from different allelic burdens among coexisting mutations, suggesting functional interplays between mutations that drive clonal selection. Based on an extensive serial/multi-regional sampling analyses, Dr. Ogawa's work further revealed high degrees of temporal/spatial heterogeneity generated during tumor expansion and relapse, which should be shaped by complex, but ordered processes of multiple clonal selections/evolutions.

Dr. Radhakrishna Pillai from the Rajiv Gandhi Center for Biotechnology presented recent findings showing that a small fraction of drug-resistant cells, named “persister cells,” may contribute to the tumor recurrence via contributing to tumor heterogeneity. Using experimental models, Dr. Pillai shared data demonstrating that persister cells remain quiescence and rarely enter into the cell cycle. However, such cells exhibit tumor stem cells characteristics and eventually lead to cell population with increased heterogeneity with respect to cell growth, invasion, response to drugs and tumor initiation potential. The team has further subjected diverse cell populations to RNA sequencing to understand both the passive and driving pathways that might be involved in drug-escape and re-emergence. The team used stable cancer cells expressing redox sensor and presented data showing that the emergence of drug resistant cells was accompanied by chronic autophagy followed by Parkin-dependant mitophagy and increased heterogeneous cell populations. Dr. Pillai suggested that such persister cells are likely to counteract the beneficial effects of chemotherapy and radiotherapy and present development of approaches to prevent emergence of drug-resistance cell populations.

Dr. Rakesh Kumar from GW Washington University presented a plenary lecture highlighting the lessons from RNA-sequencing of breast cancer sub-types in identifying the hub networks and potential new regulatory molecules. This approach re-emphasized the notion of genomic heterogeneity which, at-least, in-part, might be arising from differential splicing and promoter switching [3]. These earlier studies allowed the team to recognize the limitations of such an approach as most breast cancer sub-types are clonal in origin. To experimentally address the issue of genomic heterogeneity, Dr. Kumar laboratory decided to define the HER2 transcriptome as an example by generating isogenic MDA-MB-231 and MDA-MB-468 breast cancer cells stably overexpressing HER2. To start revealing the nature of HER2-regulated genes globelly, the team subjected such isogenic clones to microarray and RNA-sequencing approaches. Results from the microarray study identified HER2-influenced genomic elements shared among two cell lines, some of which also overlapped with that of patient microarray datasets [4]. In addition, Dr. Kumar presented scientific highlights pertaining to global transcriptomic changes and differential splicing of HER2-overexpressing isogenic clones as revealed by RNA-sequencing – a project completed in July 2014 but yet to be summarized in a manuscript by Ms. Prakriti Mudvari as a part of her dissesration research work with Dr. Kumar [5]. Following the leads from mRNA-validation studies by Ms. Mudvari, Dr. Kumar also presented preliminary data about the expression of two newly identified HER2-regulated proteins: ABCC3 with an established role in therapeutic resistance and a splicing factor with role in transformation. Interestingly, HER2-regulated ABCC3 protein with a putative role in therapeutic resistance was found to exist as multiple isoforms, raising the possibility of protein isoform-specific functions in therapeutic resistance. Further, these newly identified HER2-regulated molecules are highly overexpressed as well as co-overexpressed with HER2 in breast cancer and cancer, at-large, in genomic databases. Dr. Kumar's also discussed another HER2-centered collaborative study involving HER2 ChIP-sequencing to determine the genomic effects of the nuclear HER2 in stimulated breast cancer cells and how this will help to broaden the HER2 transcriptome. He closed his presentation with a working model to understand the molecular basis of co-overexpression and upregulation of these newly recognized HER2-target proteins with functions in drug-sensitivity and transformation, and perhaps, in HER2-biology at-large.

### Tumor Biology

Dr. Makoto Noda from the Laboratory for Malignancy Control Research at Kyoto University Graduate School of Medicine presented a plenary lecture on RECK: a tumor suppressor downregulated by multiple oncogenic pathways. Dr. Noda discussed the biology of RECK tumor suppressor which encodes a membrane-anchored glycoprotein with an ability to regulate matrix metalloproteinases. Accordingly, viable Reck–deficient mice was found to be prone to spontaneous tumorigenesis while forced RECK expression leads to inhibition of angiogenesis, invasion, and metastasis in model systems. Clinically, RECK downregulation in cancer correlates well with a poor prognosis. Dr. Noda believes that RECK provides a unique opportunity to reactivate the dormant RECK in cancer cells with a goal to suppress malignant phenotypes.

Dr. Luis Costa from the Institute of Molecular Medicine and Medical Faculty of Lisbon University highlighted the significance of molecular understanding of distinct biologic steps until the formation of clinical detectable metastases in selecting cancer patients at higher risk for metastases, and also debated if bone metastasis is a useful paradigm to follow for developing effective therapeutics. Dr. Costa presented data showing overexpression of Bone Metastases (BM) genes in tumor cells residing in BMs - independent of primary tumor type - and provided support to the cooperative function among IL11/CTGF, IL11/ADAMTS1, CTGF/CXCR4, CTGF/ADAMTS1, and MMP-1/ADAMTS1, in the bone microenvironment [6]. Dr. Costa's team shared data about the correlation between the amplification and expression of Metadherin (MTDH) which contains a lung vasculature-binding domain, and lung metastases of colorectal cancer [7]. For the first time, Dr. Costa's work showed that high MTDH expression is an independent factor for lung-relapse and survival and could be a potential biomarker for lung relapse in colon cancer. Finally, Prof. Costa stressed the need to expend the lessons of organ-specific colonization from bone metastases to other common sites of metastases, and provided a peek into very interesting set of experiments in his laboratory wherein the team is undertaking steps to capture the dynamic of gene expression during consecutive episodes of metastases in the same patient.

Dr. Dominique Scherer from the German Cancer Research Center, Heidelberg shared her vision of pharmacogenetics in colorectal cancer prevention using non-steroidal anti-inflammatory drugs (NSAIDs) which inhibit prostaglandin synthesis by targeting COX-enzymes. Although recent meta-analyses showed the effect of NSAIDs on primary cancer prevention, metastasis prevention and patient survival, the risk of gastrointestinal toxicity among NSAID remains high. Dr. Scherer presented data showing that genetic variation in specific alleles in distinct genes including PTGS1, PTGS2, PGDH and UGT modifies the beneficial effects of NSAIDs in colorectal cancer and may be also important in colorectal cancer predisposition. Her data showed that genetic variants in PGES, CRP, SRC and GPX3 were associated with increased risk of cardiac symptoms/toxicity among NSAID users. Therefore, it is crucial to identify genetic variants that interact with NSAIDs in colorectal cancer prevention and drug-related toxicities to establish targeted chemoprevention strategies.

Dr. Norman Lee from the George Washington University presented data about potential molecular basis of population disparities of prostate cancer risk with a particular focus on differential methylation patterns, expression of splicing factor and variants. The team found population-based differential alternative splicing of several key oncogenes in specimens from of African American (AA) with non-AA patients and generated variants tend to exert a strong oncogenic phenotype. Further, this phenomenon correlated well with race associated methylation patterns and expression of splicing factors. Prof. Lee hypothesize that certain splice variants specific to AA population encode gene products with a stronger oncogenic activity and thus, could explain, in-part, the noted risk of prostate cancer in certain population.

### Breast Cancer Genomics

Dr. Hisataka Sabe from the Department of Molecular Biology, Hokkaido University Graduate School of Medicine discussed the significance of mesenchymal machinery in metastasis in different cancer sub-types with a particular focus on the small GTPase Arf6 signaling pathway. Dr. Sabe presented data showing that Arf6 interacts with AMAP1 which in-turn, binds to EPB41L5 – a component of mesenchyme – and hence, contributes to mesenchymal properties that might be cancer-type specific. Because AMAP1-EPB41L5 interactions regulate E-cadherin and β1 integrin signaling, Arf6 is believed to play a mechanistic role in epithelial-mesenchymal transition. Dr. Sabe also presented data showing the activation of Arf6 by two distinct mechanisms – by the cell surface tyrosine kinases in breast cancer and by G-protein coupled receptors (GPCRs) in clear cell renal cell carcinomas. Therefore, Arf6 activation - independent of its upstream mode of stimulation-cooperates with the Arf6-AMAP1-EPB41L5 pathway to promote EMT. Dr. Sabe closed his presentation by highlighting the roles of genome in eliciting the Arf6-driven EMT pathway in metastasis of human cancer.

Dr. Sudeep Gupta from the Tata Medical Center discussed a recently conducted randomized study wherein a single depot injection of progesterone prior to surgery in patients with node positive breast cancer exhibited clinical benefits [8]. Dr. Gupta shared results from on-going studies designed to understand the potential mechanisms behind the noted effects of progesterone. Using cell-based model systems, the team performed genome wide expression analysis and identified serum and glucocorticoid regulated kinase SGK1 as a progesterone up-regulated transcript. In addition, progesterone also down regulated two microRNAs that target the 3′UTR region of SGK1. Dr. Gupta proposed a novel dual-regulatory model wherein progesterone up-regulates SGK1 as well as down-regulates the expression of microRNAs which in-turn, targets SGK1. He also suggested that progesterone could downregulate the expression of its own receptor through upregulation of a microRNA.

Dr. Louis Chow from Organisation for Oncology and Translational Research and UNIMED Medical Institute presented a comprehensive comparative summary of available diagnostic gene signatures and assays for breast cancer prognostic and/or predictive assessment. Dr. Chow highlighted the benefits of including the proliferation or estrogen receptor (ER) pathway in assay platforms for prognosis. For example, the 21-gene Oncotype DX assay is a prognostic and predictive test that uses ER, proliferation, HER2 genes, amongst others to normalize for RNA quantity, and the expression levels are used to calculate a recurrence score for ER positive, node negative breast cancer. In contrast, Endopredict uses 12-genes not involving ER, PR or HER2 status to classify the prognosis of HER2 negative breast cancer. The gene signatures also predict the risk of recurrence for patients - high probability of metastasis, free survival, or high risk for distant metastasis.

### Targeted Cancer Therapeutics

Dr. Stefan Knapp from the University of Oxford presented an update on recent development of chemical probes for epigenetic reader domains of the bromodomain family. Bromodomains (BRDs) are evolutionary conserved protein interaction modules that specifically recognize ε-N-lysine acetylation motifs, a key event in the reading process of epigenetic marks. The human proteome encodes 61 of these highly diverse domains present in 41 mainly nuclear proteins. Recently, Dr. Knapp's research group has developed a platform for screening and designing of specific bromodomain inhibitors based on a large number of crystal structures and biochemical/cell based assays [9]. The probe project at the SGC (http://www.sgc.ox.ac.uk) resulted in the development of a comprehensive set of chemical probes covering most the human bromodomain family. The development of these research tools led to new validation of new targets and the initiation of first set of clinical studies in cancer [10, 11]. As an example of the translational aspects of this effort, Dr. Knapp reported on the development of dual kinase/bromodomain inhibitors [12] and specific chemical probes targeting the bromodomains of CBP/P300 [13] and BRPF1B [unpublished].

Dr. Junko Murai from the Medical Innovation Center at the Kyoto University Graduate School of Medicine presented new research revealing a novel mechanism of action for poly(ADP-ribose) polymerase (PARP) inhibitors – the most promising anti-cancer agents. The mechanism of anticancer effect of PARP inhibitors has been interpreted by single strand break (SSB) repair inhibition through catalytic PARP inhibition. Her team discovered that inhibitors promote trapping of PARPs at the damaged DNA site and that trapped PARP-DNA complexes are more cytotoxic than unrepaired single-stranded breaks [14]. Dr. Murai also found that the potency of PARP-trapping is markedly (more than 1,000 folds) different among five clinical PARP inhibitors in-spite of an equivalent high catalytic PARP inhibition [15]. Dr. Murai stressed the need to start considering the PARP-trapping efficacy as an addition variable for clinical utility of a given PARP inhibitor.

### Personalized Medicine

Dr. Stephen Fox from Peter MacCallum Cancer Centre in Melbourne shared his vision of “Cancer 2015 - a model for personalized medicine” program. Cancer 2015 study is a large-scale, prospective, multi-site cohort of newly diagnosed cancer patients from Victoria, Australia with 1718 patients recruited, 854 patients of which have been currently sequenced for 48 cancer genes. Clinically relevant mutations were identified in 63% of patients, with 26% of patients displaying a mutation with therapeutic implications. While the prevalence of mutations was consistent with other institutionally based series for some tumor types (breast carcinoma and colorectal adenocarcinoma), others were different (lung adenocarcinoma and head and neck squamous cell carcinoma) which has significant implications for health economic modelling of particular targeted agents. Further, in some tumour types new areas of research were identified through recognition of novel genotypes. Reliable delivery of a diagnostic assay to screen for a range of actionable mutations was achieved, providing evidence for a re-classification of some cancers and opening unexpected avenues for investigation and treatment of cancer patients. Dr. Fox also presented his vision about the planned utilization of the Cancer 2015 study for modeling health economics of particular targeted agents.

Dr. Fumihiko Matsuda from the Center for Genomic Medicine at Kyoto University Graduate School of Medicine presented the utility of a large-scale genome for comprehensive human biology. As a model of “Human Biology”, the team initiated “The Nagahama Cohort Study” - a community-based prospective genome cohort study in Nagahama City. The baseline survey started in 2007, and the enrollment of 10,082 healthy residents was completed in 2010. In addition to the collection of health-related information and the measurement of 115 biochemical and hematological traits, the team conducted an integrated comics study. A genome scan using SNP arrays was initiated in 2009, and approximately 4,000 DNA samples have been analyzed to date. Dr. Matsuda presented interim analysis of overall data and discuss the results of the cross-sectional analysis.

## CONCLUSIONS

The symposium achieved one of the core GCGC goals to bring together its founding members from six institutions as well as a large number of interested scientists and potential collaborators to share their exciting research findings. Looking at the trend during the past meetings, it was obvious that certain research themes such as tumor heterogeneity and transcriptome have evolved over the years. It remains unclear whether we are able to harvest the fruits of whole genome sequencing completed so far. In general, each session provided a spectrum of findings ranging from an overview to specific research questions that might be acting as a bottleneck to move the field forward. As always the case in science and medicine, many of the presentations raised more exciting questions for investigation while addressing a well-defined research problem. The organizing committee as well as some of meeting participants observed the need to include additional shared activities some of which might require financial resources. The group identified a short list of broad activities that might be feasible within the current framework of consortium members and the precise nature of such collaborative theme is likely to be evolved soon.
